# Burnett’s Disinfecting Fluid

**Published:** 1847-11

**Authors:** 


*Burnett's Disinfecting Fluid.*—The chloride of zinc in solution, it appears from a parliamentary document which has just been issued, has been employed extensively as a disinfectant in dissecting rooms,the wards of hospitals, and in the royal navy, and, according to the reports which we have seen, has been eminently successful in effecting the objects for which it is designed. The medical offieers at Haslar Hospital state that it has been used in that hospital in the close stools of patients affected with dysentery, in the water-closets and cesspools, and also in the wards, when the air was tainted by purulent expectoration or discharge from sores, with the effect of immediately removing the disagreeable odours. It has also been used in surgery with good effect, in removing the smell of putrefying animal substances, and the odour of dead bodies under inspection: when employed as a dressing to ulcers, it removes the disagreeable smell of purulent matter, and, in the proportion of one part of the clear solution to eighteen of water, it preserves subjects of natural history from putrefaction, and in a fit state of anatomical inspection, after more than a year has elapsed. A similar testimony in favor of the solution of the chloride, is borne by the assistant surgeon of the Marine Hospital at Woolwich, who adds, "the great advantage which the chloride of zinc possesses over other agents employed for a like purpose, is, that it removes the disagreeable effluvium, without leaving one little less offensive in its room, and may therefore be made use of wherever this effect is required —in private as well as public buildings, in the sick bed chamber no less than in the crowded ward. The method adopted at this hospital is to supply each of the wards with a bottle of the diluted solution, which the nurses have directions to use whenever occasion may require, besides sprinkling it over the floors before the morning and evening visits are made.

Its utility in the dissecting-room is confirmed by the statements made by Mr. Bowman, Dr. Sharpey, Mr. Patridge, Dr. Murray, and Dr. V. Peltigrew, who concur in asserting, that in a proper degree of dilution its success is complete, and that it appears to preserve the color and texture of the parts very admirably. It has, futher, the very important advantage of not acting on the steel instruments employed, being in this respect equal to alcohol. Dr. Methven especially mentions an instance in which the solution corrected advancing putrescence, and enabled him to dissect during July. He believes, further, it will be the means of saving many valuable lives, which are annually lost by wounds received in the course of dissection, as, while dissecting this putrid body, he cut himself several times, and once received a punctured wound, without any bad consequences arising. Mr. M'Bain, of the "Mastiff," adds his testimony "to the rapid and perfect effects of the chloride of zinc solution upon animal matter in a state of putrefaction. Having frequently opportunities of dissecting or examining large fish, &c., cast on shore, whilst undergoing decomposition, the task has been occasionally any thing but agreeable, for want of a convenient power to destroy the putrefactive process. The chloride in these cases acts like magic; and as a great practical agent over one of the most import conditions of animal and vegetable matter—viz: putrefaction, it stands unrivalled." Its influence on board ship, in annihilating the offensive smell of bilge-water, and in sweetening between decks, is shown by the united evidence of captains, surgeons, and masters in the royal navy. Among other vessels, it was used on board the "Victoria and Albert" royal yacht, to remove a more than ordinary stench of bilge-water, and other offensive odors, with the most complete success. The surgeon states that she has remained comparatively sweet ever since, and when a bilge-water smell is occasionally perceptible, a slight application of the fluid removes it. The solution has also been used for very disgusting privies, &c., effluvia from which, it quickly neutralizes.—*London Lancet.*

